# An exploratory qualitative study of life trajectories from preschool-age to young adulthood: Identifying early biologic sensitivity, facing challenges and moving forward

**DOI:** 10.24105/apr.2018.5.8

**Published:** 2018

**Authors:** Abbey Alkon, W. Thomas Boyce, Aaron Shulman, Roberta S. Rehm

**Affiliations:** 1 Department of Family Health Care Nursing, UCSF School of Nursing, CA, USA; 2 Division of Developmental Medicine, UCSF Department of Pediatrics and Psychiatry, CA, USA; 3 Idea Architects Literary Agency, 116 Oxford Way, Santa Cruz, CA, USA

## Abstract

**Background:**

This exploratory qualitative study explored the life experiences of young adults who participated in a cohort study in their child care center 26 years ago.

The purpose of the study was to: (1) Describe the life trajectories of study participants who exhibited the extremes of high or low cardiovascular reactivity during their preschool ages. (2) Identify the life courses, processes, or outcomes for these young adults. (3) Describe exemplar cases of children with high and low reactivity who illustrated patterns of resilience or vulnerability.

**Methods:**

Eight out of the 137 children who had combinations of extreme high or low reactivity and environmental adversity were identified and interviewed by a blinded researcher. Data were analyzed through iterative coding, development of major categories, matrix analysis and thematic analysis.

**Results:**

The overall theme for all of the participants was facing challenges, and moving forward. The major categories which showed some variation between those with high and low reactivity were developing sources of support, overcoming adversity, and finding satisfaction/ dissatisfaction with life.

**Conclusion:**

These life histories provide a further understanding of how biologic sensitivity to challenges identified early in life may have impacted participants’ trajectories from preschool to young adulthood, and indicate that further study would be warranted across the life course.

## Introduction

Young children’s biological sensitivity to stressful situations can be either a vulnerability or resilience factor for developing physical and mental health problems later in life [1–4]. Young children who have high biologic sensitivity and grow up in stressful environments are at risk of developing aggression or externalizing behavior problems [2,5]. On the other hand, young children who have high biologic sensitivity and grow up in low stressful environments, including nurturing and sensitive parenting, are at a low risk of developing behavior problems [6,7]. Children’s biologic sensitivity to positive and negative life experiences develops during sensitive periods of development and gets embedded into their physiology [8]. The biological sensitivity to context (BSC) theory [9] states that individuals differ in their bio-behavioral reactivity to the environment and these individual differences in reactivity modify the relationship between early childhood, familial, and environmental exposures, such as maternal depression [5], crowded housing [5], maternal sensitivity [10], father involvement [11], and future mental and physical health problems. These groundbreaking studies using the BSC theory have extended our understanding of the influence of early life experiences, biologic sensitivity, and health but they do not explore the qualitative individual differences in life course for children with high and low biologic sensitivity and how it could affect their development from early childhood to young adulthood.

Stressors have been defined as events or conditions that threaten, or are perceived to threaten, physiological equilibrium [12]. Some stressors experienced early in life or within the family, including maternal depression, lack of maternal sensitivity, living in poverty, household crowding, trauma, cultural assimilation, health problems, and parental separation, can negatively impact children’s development. Physiologic responses to stressors involve central nervous system activity that mobilize endocrine, autonomic, and behavior systems to protect from and/or adapt to a threat [13]. The stress response system primarily involves the sympatho-adrenal system (SAM) and hypothalamic-pituitary-adrenocortical (HPA) axis.

The stress response system has three main biological functions: (1) to respond to physical and psychosocial challenges; (2) to identify and filter information about the social and physical environment; and (3) to regulate physiology and behavior in response to the environment [14]. The calibration of the stress response system depends on the encoding of a child’s experiences early in life and the environmental and socio-emotional feedback experienced. Individual differences in responding to these stressors emerge early in life and over time. In most studies the prevalence of extreme physiologic responsivity is approximately 15% of the sample [15], thus, the majority of children develop adaptive patterns of responsivity.

Reactivity is defined as the change in a person’s physiology from a resting state to a challenging condition. Children with a high biologic reactivity show increased blood pressure (BP) during a challenge compared to lower BP during a resting or basal condition. Children with hyporeactivity or low reactivity (LR) show little increase, or a decrease, in BP during the challenge condition compared to the resting condition. Low reactivity may be a sign of chronic psychosocial stress where limbic regulatory pathways are blunted [13]. Children with low reactivity may be demonstrating a degree of unemotional responsivity or callousness that may be adaptive under certain circumstances. Children with high reactivity show a large increase in BP during the challenge conditions compared to the resting condition. Increased reactivity activates the threat response system which increases corticotropin-releasing hormone (CRH) activity in the central nucleus, remodeling the dendrites of the amygdala.

Cardiovascular reactivity in response to an emotional or stressful stimulus can be adaptive and facilitate children’s ability to manage their emotions and behavior. In a study of boys 5 to 13 years of age, children with high respiratory sinus arrhythmia (RSA) reactivity, a measure of the parasympathetic nervous system, had more adaptive emotional regulation responses and lower rates of depression than children with lower RSA reactivity [16]. In a study of young adults with high parasympathetic nervous system (PNS) reactivity, they demonstrated more socially adaptive emotion-regulation strategies than young adults with lower PNS (i.e., RSA) reactivity [17]. The high reactive young adults used coping skills, were more socially engaged, and utilized social support when dealing with distress and sadness. When angered by others, they were more likely to make concessions compared to their lower reactive peers. In several cohort studies, young adults with low reactivity had mental health problems and unstable relationships later in life, such as delinquency [18] and unsatisfying romantic relationships. Although these cohort and cross-sectional studies found reactivity was related to emotional or behavioral problems, none of the studies measured reactivity when the participants were preschoolers or included qualitative interviews to explore the participant’s individual perspectives on their life course.

### Framework and theory

The biodevelopmental framework demonstrates how exposure to early adversity along with biological embedding during sensitive periods of development affect a child’s physiologic adaptation and responsivity to predict adult outcomes in learning, behavior, and health [19]. The preschool-age is a sensitive period when young children exhibit plasticity and develop formative physiologic responsivity to environmental challenges in the face of protective factors, such as maternal sensitivity, stable relationships, and genetic predisposition. The life trajectory of young adults is complex and there are many time points and experiences along their path to adulthood that can support their success or contribute to their challenges [20].

The purpose of this exploratory qualitative study was to: (1) Describe the life trajectories of a small sample of young adults who exhibited the extremes of high or low cardiovascular reactivity and were exposed to high and low levels of stress in child care during their preschool ages, (2) Identify the family relationships, life courses, processes, and outcomes for these young adults, and (3) Describe exemplar cases of the young adults who had high and low reactivity as preschoolers and who illustrated patterns of vulnerability or resilience.

## Methods

The Preschool Health Project (PHP), conducted over two years (1990–1992) in four child care centers, identified children’s biologic sensitivity to a standardized stress protocol while monitoring their cardiovascular measures, specifically heart rate and blood pressure (BP), and environmental stress in the child care environment [15]. The PHP included 137 three to five year old children, 60 girls and 77 boys, and 75% were White. The parents had a mean education of 18 years, 86% were married, and 100% had at least one parent working outside the home. The stress protocol was administered by a research assistant to the children in a quiet, private space in their child care center without a parent present. The protocol included resting measures, calm stories read aloud, and seven challenging tasks presented in the same order for all the children: social interview, block construction, number recall, Gestalt closure task, social problem solving task, blinded object identification, and verbal description of an emotional event [21].

A blood pressure cuff was attached to the children’s non-dominant arm. Measurements of heart rate and mean arterial blood pressure (MAP) were completed using an automatic, oscillometric Dinamap monitor (model 1846 SX/P, Critikon, Inc., Tampa, FL). The BP cuff was inflated at a standard point in the presentation or completion of the resting condition or challenge. The MAP is the point of maximal oscillatory amplitude which can be accurately ascertained in children with relatively small differences between systolic and diastolic pressures. The MAP reactivity was classified as high reactivity or low reactivity based on the difference between the mean MAP during the challenge conditions and the mean of the MAP during the first resting condition.

The measure of environmental stress in the child care environment was quantified as an index of the overall child care quality using five components of the child care environment [22]: a standardized overall child care quality measure, the Early Childhood Rating Scale (ECERS) [23] completed by a trained researcher. The ECERS included measures of space and furnishings, language-reasoning, personal care routines, activities, and teacher-child interaction. The index also included four demographic characteristics of child care center environments related to quality care: staff to child ratios, ratio of full- to part-time staff, level of staff education, and staff turnover rates. Studies show that low levels of teacher education and high staff turnover contribute to lower quality centers that have more stressful environments than high quality centers [24–26]. The ecologic, environmental stress index was quantified by standardizing each of the five variables and then creating a summary index.

### Recruitment and sample

Eight participants from the original sample of 137 were selected to participate in this follow-up, qualitative study based on maximal variation sampling of reactivity and stress exposure histories, not demographic characteristics, identified in the PHP study. Using maximal variation sampling, a small number of cases were selected in order to document variations in response that have resulted from adaptation to different conditions, and to identify common patterns that occurred across the varied circumstances of participants [27]. Participants were chosen because their histories placed them in one of four categories (2 participants for each category): (1) High reactivity/high exposure to environmental adversity, (2) High reactivity/low exposure to environmental adversity, (3) Low reactivity/high exposure to environmental adversity, and (4) Low reactivity/low exposure to environmental adversity [15].

Investigators had no contact with the participants after the original study was completed until the selected participants were asked to participate in the follow-up study. We initiated contact with eight participants, two in each of the extreme reactivity/environmental adversity categories, but two participants did not respond to social media and email correspondence. Then, we identified the next two participants who had extreme high or low reactivity/environmental adversity, who agreed to participate in the study. The demographic characteristics of the eight participating young adults was: 29 to 31 years of age, five males, three females, all White, college graduates, five were American born, all living in the U.S. at the time of data collection. All of the participants were either working or in graduate school at the time of the interviews. Both the original and follow-up studies were approved by the investigators’ university institutional review board.

### Interview procedure

Interviews included initial open-ended questions, followed by semi-structured probes. All interviews were 1 to 2 hours in length and were conducted by a trained research assistant who was blinded to the young adults’ reactivity and adversity profiles. Interviews were conducted in-person (n=7) in a private location, such as an apartment or office space, or by skype (n=1). The interviews were audio recorded and transcribed verbatim by a third party service.

Interview topics included: earliest memories, school years (successes *versus* challenges, related emotions, outcomes), interests and passions (settings and pursuits where young adult feels most engaged and happy, reflections on why), career trajectory (successes *versus* challenges, related emotions, outcomes, future goals), family and personal relationships (family, friends, romantic partners; successes *versus* challenges in maintaining these relationships), and self-reflection (thoughts or awareness on how physiologic reactivity affects his/her behavior, strategies learned to guide their life decisions). The interviewer wrote field notes to summarize the interviews, context, and his impressions immediately after each interview.

### Analysis

Data analysis included a multi-step process. The first and last author were blinded to the participant’s reactivity profile and they read all transcripts to get an overall sense of the data, then independently engaged in open coding [28]. Initial open codes were discussed, combined and developed into salient categories used in a subsequent, independent, matrix analysis using these categories. Matrix analysis is a form of focused coding in which categories are linked to relevant data to assure that categories are fully developed and faithfully capture variations and commonalities across participants [29]. In the matrix we used categories developed from open coding on the top axis and entered data for each participant, followed by placing data into the reactivity/stress exposure cells which each participant represented. Matrix analysis grids were compared across investigators, and together we performed a thematic analysis to identify the major categories of findings and an overarching theme [30]. This careful, comparative analysis allowed us to identify both commonalities and differences in life experiences of participants and to identify exemplar cases of differences by biologic sensitivity.

## Results

The overarching theme was “facing challenges and moving forward” and was developed from three major categories constructed to capture the important aspects of the participants’ lives: (1) supportive relationships, (2) facing adversities, and (3) satisfaction with current situation. There were some variations between high reactive (R) and low reactive (LR) participants within these categories; however, the overarching theme of “facing challenges and moving forward” was applicable to all participants, regardless of reactivity levels. Table 1 summarizes the categories that the eight participants represent: high and low MAP reactivity and high and low child care adversity.

The participants were at the extremes of the reactivity continuum, either high or low reactivity, and they all faced challenges and were moving forward in the course of childhood, youth, and young adulthood. All of the participants had sustained at least one supportive relationship, had graduated from college, were financially independent, and continued to acknowledge and face challenges, and found satisfaction in at least some aspect(s) of their lives.

**Supportive relationships** were described with family members, peers, and/or romantic partners. All of the participants identified supportive relationships with different persons, length of support, and types of support. Participants often described many of these relationships as long-term and ongoing. Types of support described included emotional, social, recreational, and material sustenance. Participants also described conflictual relationships with family, friends, and partners through the years, but all maintained current active supportive relationships at the time of the interviews.

Family relationships were described as supportive and understanding by most of the participants yet one participant said his parents did not talk about their feelings (ID 200 – R) and two other participants (ID 400 – LR, ID 301 – LR) said their parents did not talk about sensitive issues. One participant explained how his family dealt creatively with his frustration at the family’s dinner table:

“At one point, they (parents) got a talking stick that they would pass around the table to just sort of control the conversation because I was flipping out so much.’ (ID 101-R).

The long term nature of many participants’ relationships was expressed by one participant, who said:

“There’s like three or four of us that have known each other since preschool and have gone on through kindergarten, elementary, middle, and high school together” (ID 200-R).

Participants described a history of making friends that began in school and extended to young adulthood. One said, “I was really happy with my social mix in high school for most of it” (ID 300-LR). Another participant took longer to make friends, and said: “It would take me a really long time to adjust and get to know people but then, once I did make friends, I was fine” (ID 301-LR).

One participant focused more on the present relationships at work compared to school and said, “I make pretty good connections at work, I’m close to people I work with” (ID 100-R). This participant was the only one who had no, long term romantic partner. The participant said:

“I’ve never really been in love. It’s just… I don’t know. I normally don’t get attached that way I guess. I’m totally all right being a loner” (ID 100-R).

**High reactivity (R) versus low reactivity (LR).** It was noteworthy that three out of the four reactive participants said they liked time alone (ID 100-R, ID 101-R, ID 201-R), yet none of the low reactivity participants mentioned time alone. Two out of the four reactive participants (ID 201-R, ID 200-R) identified influential adults who were formative role models and supported their development in high school. Yet, two out of the four low reactive participants mentioned that their families didn’t talk about sensitive issues (ID 400-LR and ID 301-LR).

The two extreme reactive participants had opposite experiences with romantic relationships and this could be attributed to the interaction shown in research studies. Reactive participants are extremely sensitive to environmental stress and depending on the type of family support they experienced early in life they had different romantic relationships. For example, one reactive participant who described his family as sensitive to his emotional needs had a strong, long term romantic partner yet the other reactive participant who described his family as not emotionally sensitive, acknowledged that he had a history of excessive drinking, and had no long term romantic partner.

**Facing adversities** includes challenges experienced in school, such as being bullied or academic difficulties, or general life’s challenges, including the family, culture, relocation, legal problems, and death. All of the participants faced life challenges which caused distress either temporarily, or on an ongoing basis. Examples of challenges included unsupportive parents, struggles to define sexual identity, instances of childhood bullying, relocation to and from other countries over the years, abuse as an adult, shoplifting, and parental and other family deaths. In some instances participants took active steps to confront and mitigate these challenges, but others resolved their challenges over time or were ongoing. For example, a young woman faced multiple challenges while she was in college. She said:

“There is a big thing that I didn’t mention, I gained a lot more weight in college … my parents offered to enroll me in a … weight loss thing which I did” (ID 300-LR).

This participant lost much of the weight gained, but faced another major challenge: “I was arrested once for stealing…. it was pretty traumatizing.” This participant spent several years meeting the terms of the sentence and eventually managed to have the conviction expunged; however, the participant’s criminal record continued to surface in employment and school endeavors, and the participant showed her maturity and transparency by creating a strategy for discussing and explaining it to mentors and employers.

One participant faced personal adversity yet maintained everyday routines to stay in graduate school. The participant said: “My roommate … committed suicide … so that was terrible” (ID 400-LR). This participant mourned the loss of the roommate while maintaining a rigorous schedule in graduate school.

Another participant recounted a family death that took many years to process because the family did not discuss it or actively support each other:

“My dad passed away when I was in high school … I think everyone just shut down in my family; it was really hard because … everyone got depressed in their own space and we just never really talked about it” (ID 301-LR).

Participants often overcame adverse events from childhood and many of them remembered the events vividly. For example one participant recalled,

“I was taking public transportation to and from (middle school) and I was going through a real awkward phase where I was starting to sort of become a target for bullies” (ID 101-R).

This participant “found his tribe” when he discovered acting and joined the drama program at his high school where he made friends and began to fit in.

Another young man described several challenges:

“I wasn’t focused (in school), I didn’t learn to read as fast as other people. I don’t remember, probably third grade is when I started to. I think the school wasn’t very accepting of that apparently. Again my parents didn’t really react” (ID 201-R).

This participant went on to be very successful professionally, but described troubled relationships with his parents and drew most of his support from friends.

**High reactivity (R) versus low reactivity (LR):** The high and low reactive participants showed some differences in overcoming adversity. Two out of the four high reactive participants experienced being bullied and picked on earlier in life but none of the low reactive participants had these experiences.

Two out of the four high reactive participants mentioned watching television to relax yet none of the low reactive participants mentioned television. Two out of the four low reactive participants experienced trauma around friendships that were not healthy and they noted that they ‘followed others’, ‘let others make decisions’ (ID 300-LR), or were ‘blind to it’ since they didn’t realize the consequences of their action (ID 401-LR).

**Satisfaction with their current situation** at work, with housing, or a relationship was discussed with the participants. All of the participants could identify some areas of great satisfaction in their present lives, such as a satisfying career or positive romantic relationship.

“I grew up in an environment where I could achieve highly in a way that … In many ways felt satisfied or felt as nearly satisfied that thrived in my professional life” (ID 400-LR).

“I was an achiever and that was the way I defined myself I think, and so it was great. They (his parents) were very proud of that and doing exactly the kinds of things they would want me to do …” (ID 300-LR).

“Theater’s just made such a huge difference for me. It’s made me such an outlet. I know where to meet people and it still remains artistically satisfying” (ID 101-R).

Several participants also described specific dissatisfactions with their current life situation, which they characterized as guilt, frustration, or restlessness. Five of the eight participants sought therapy to help them cope with their struggles in high school (2 participants) or present day circumstances. One participant said:

“It was really difficult I think (transition after college). “I’m still adjusting because I graduated in 2010 (recession), things were getting better but it was still hard… I’m having a hard time reconciling my childhood which was full of all these opportunities” (ID 200-R).

Another participant was restless about his present situation and said:

“The people here are so boring. First of all with this weather, it’s dirty for a small city. It’s so many homeless people, … so homogenous, and it’s a very segregated city, it’s not an international city…” (ID 201-R).

A low reactive participant shared a similar thought as a reactive participant about feeling privileged growing up but not totally satisfied as a young adult.

“I actually think I still do have some emotional baggage, kind of not feeling cool or not feeling like confident that way I wanted to be” (ID 400-LR).

**High reactivity (R) versus low reactivity (LR).** There are some specific experiences that exemplify differences between preschoolers with reactive *versus* low reactive profiles. Two of the four reactive participants who were employed and had romantic partners expressed discontent with their current situation. None of the low reactive participants expressed similar levels of discontent. One participant with high reactivity was a highly successful businessman with a long term girlfriend; however, he disliked his city of residence, acknowledged being unfaithful to his girlfriend, and wanted to relocate to another city after assuring his financial stability (ID 201-R). Another participant with high reactivity was an architect and employed in his field, but dissatisfied with his salary and the high cost of living. At the suggestion of his long-term girlfriend, he had recently started therapy to cope with these feelings (ID 200-R).

Participants who were less reactive had ongoing challenges but they were more likely to express their ability to deal successfully with their struggles and move on with their lives. For example, a participant had been arrested for shoplifting as a college student and continued to encounter instances when this record was exposed to employers, despite an official expungement. Nevertheless, she enrolled in graduate school and was determined to proactively disclose this incident to ward off professional trouble in the future (ID 300-LR). Another participant with low reactivity who experienced an abusive work situation dealt with the situation by moving to a new city and finding a stimulating job in her area of technical expertise. She could express her continuing goal to strive and said “I still don’t know what I want to be when I grow up.” Nevertheless, she said. “I like it (job in new city); it’s fun and my co-workers are really great” (ID 301-LR).

There were two reactive participants who exemplified the different trajectories in life depending on their early life experiences. High reactivity children are more vulnerable than low reactivity children and their future mental and physical health may depend on their ability to deal with the challenges in their lives. Some of the known protective factors that support the high reactive children to positively cope and show resilience under conditions of adversity are maternal sensitivity, supportive relationships, coping strategies, or emotional regulation [6,31,32]. One of the reactive participants experienced bullying and depression as a school-age child but lived with a very supportive family who found creative ways for him to express himself at home. By high school, he found a new home in the theatre world and established a successful career for himself along with establishing a long-term romantic relationship (ID 101-R). On the other hand, another reactive participant identified being bullied in elementary school and having a lack of emotional family support. He has one long-term close friend from high school and never had a romantic partner for more than a few months. He has a successful career path and is satisfied with his work environment and work colleagues yet he drinks alcohol daily, often to excess, and lives in his parent’s house (ID 100-R). Although both reactive participants faced childhood adversities, their families’ support was very different which may have affected their ability to develop positive (or negative) coping mechanisms and romantic relationships.

There are two low reactivity participants who exemplify the life course for children who do not physiologically respond as strongly to the environmental challenges that they encounter. In some research studies these children thrive in both high and low stress environments [15,33] since they may not be responsive to the environmental cues.

One low reactive participant was in a negative work environment but couldn’t confront the abusive boss or share her experience with family members or friends. She removed herself from the situation by abruptly quitting the job and moving to a new city. Although she finds it easy to meet new friends, they are not always healthy friendships. Her resilience under stressful conditions is evident, yet her actions do not reflect the introspective or active confrontation of challenges identified by some reactive participants. She is able to continue with her work and social life even under difficult circumstances. She is very positive about her future career and romantic relationship with no regrets or remorse (ID 401-LR).

Another low reactivity participant also makes friends easily but allowed herself to be led into a dangerous situation by a close friend. They both were arrested after shoplifting and had a traumatic experience in jail. She has been very positive about the future and is able to move on with her career goals, romantic relationship, and is forthcoming about her arrest record with future employers. (ID 300-LR). Both of these low reactivity female participants (ID 401-LR, ID 300-LR) found themselves in risky situations, allowing men to make decisions that affected their lives but they took actions that showed they could move on with their lives. They do not appear to linger in these traumas but quickly change their circumstances to pursue their goals. They are both in stable romantic relationships, have steady jobs, and are pursuing their careers.

## Limitations

Although this study is novel in its design and purpose, there are several limitations that should be addressed. This purposive sample included only eight young adults and they may not represent the extreme reactive and less reactive children in other studies. We targeted a small sample to investigate if there were differences between children with extreme sensitivities. This sample was highly educated and employed which may account for the over-arching theme of ‘facing challenges and moving forward’. Even under conditions of adversity, young adults who grew up in primarily two-adult, employed households with educated parents are more likely to succeed compared to young adults growing up in poverty and low resource households [20]. The measures of physiologic reactivity have changed since the 1990s due to new hardware and software allowing researchers to identify specific parasympathetic and sympathetic nervous system reactivity rather than the integrated measure of blood pressure available in the 1990s. Presumably, specific physiologic measures may be more sensitive biologic measures of reactivity compared to blood pressure and thus, provide stronger findings than our original study. Another limitation is the lack of repeated interviews to validate the findings and identify more in-depth themes on mental and physical health conditions.

## Discussion

This exploratory qualitative study with a purposive sample of eight young adults who were either biologically reactive or less reactive during their preschool years showed some common overarching themes across all the young adults. Exemplar stories illustrated and supported the research showing that young children who are reactive may have different life courses depending on their supportive relationships during childhood [2,5,6]. The exemplars of less reactive children also supported the literature showing that children with low reactivity may demonstrate a level of resilience, such that they thrive under conditions of both low and high stress [2,31].

The life trajectory of these young adults who lived in urban, thriving communities showed some common experiences. The participants all attended child care centers fulltime when they were three to five years of age. Their parents were employed and financially secure. We can hypothesize that these common demographic characteristics strongly influenced their life trajectories. They developed friendships during their school-years while attending different elementary, middle, and high schools. They all attended and graduated from college and are now establishing their professional careers and economic independence. Their friendships may have changed over time but most of them had stable friendships over many years. Although seven out of eight of the participants experienced at least one romantic relationship, they differed in intensity and longevity.

Early life stressors experienced by some of the participants included a lack of parental support, being bullied by peers, or parental death which may increase their risk of future psychopathology, particularly disorders of emotion and attention regulation [13]. For example, one participant with high reactivity had a tumultuous relationship with his parents, was a hyper-sensitive child, and presently has difficulty with self-regulation and struggles with discontentment (ID 201-R). This qualitative case study illustrates the findings from larger quantitative research studies. Although the qualitative stories are not generalizable, they provide a rich narrative to understand and explain other studies’ findings.

Further study is needed to explore the adult life course outcomes for a socio-economically diverse population of children with high and low reactivity.

This study and others have shown how children as young as three to five years of age show a range of physiologic responses to environmental and emotional stressors. This wide range of responses indicates individual differences in young children’s physiologic responsivity to stressful experiences early in life that may impact their ability to cope or respond to stressors later in life. Pediatric health care providers can talk to parents and guardians about their responsivity to different children’s needs during stressful experiences. Some children are more vulnerable and need physical warmth and reassurance while others may thrive even under conditions of stress. Parent’s ability to provide a trusting, warm relationship with their child impacts their child’s ability to deal with stress. Children with high reactivity, or exaggerated physiologic responses during stress, are vulnerable but they can thrive if their parents are sensitive and responsive to their emotional needs. Health care providers, researchers, and parents can become more aware of the differences that children experience when they are stressed to be able to support them during these difficult times and enable them to cope and regulate their emotions later in life.

## Figures and Tables

**Table 1.** Classification of participants by Mean Arterial Pressure (MAP) reactivity and child care adversity.

MAP Reactivity	Exposure to Child Care Adversity	
	High levels of exposure	Low levels of exposure
High Reactivity (R)	ID # 100	ID# 200
	ID # 101	ID# 201
Low Reactivity (LR)	ID# 300	ID# 400
	ID# 301	ID# 401
